# Crowded environments tune the fold-switching in metamorphic proteins

**DOI:** 10.1038/s42004-023-00909-2

**Published:** 2023-06-08

**Authors:** Ning Zhang, Wenyan Guan, Shouqi Cui, Nana Ai

**Affiliations:** 1 Qingdao Institute of Bioenergy and Bioprocess Technology, Chinese Academy of Sciences, Qingdao, 266101 China; 2 Shandong Energy Institute, Qingdao, 266101 China; 3 Qingdao New Energy Shandong Laboratory, Qingdao, 266101 China; 4 Materials and Biomaterials Science and Engineering, University of California, Merced, CA 95343 USA; 5 University of Chinese Academy of Sciences, Beijing, 100049 China

**Keywords:** Biophysical chemistry, Solution-state NMR, Protein folding, Circadian rhythm signalling peptides and proteins

## Abstract

Metamorphic proteins such as circadian clock protein KaiB and human chemokine XCL1 play vital roles in regulating biological processes, including gene expression, circadian clock and innate immune responses, and perform distinct functions in living cell by switching different structures in response to cellular environment stimuli. However, it is unclear how complex and crowded intracellular environments affect conformational rearrangement of metamorphic proteins. Here, the kinetics and thermodynamics of two well-characterized metamorphic proteins, circadian clock protein KaiB and human chemokine XCL1, were quantified in physiologically relevant environments by using NMR spectroscopy, indicating that crowded agents shift equilibrium towards the inactive form (ground-state KaiB and Ltn10-like state XCL1) without disturbing the corresponding structures, and crowded agents have predominantly impact on the exchange rate of XCL1 that switches folds on timescales of seconds, but have slightly impact on the exchange rate of KaiB that switches folds on timescales of hours. Our data shed light on how metamorphic proteins can respond immediately to the changed crowded intracellular conditions that induced by environmental cues and then execute different functions in living cell, and it also enhances our understanding of how environments enrich the sequence-structure-function paradigm.

## Abstract (web summary)

Metamorphic proteins can switch reversibly between two different native folds with entirely distinct functions, however, the triggers that induce the conformational switch remain largely underexplored. Here, the authors demonstrate the impact of a crowded environment on the switch of two metamorphic proteins KaiB and XCL1, and study the kinetics and thermodynamics of the switch using NMR spectroscopy.

## Introduction

**Fig. 1:**
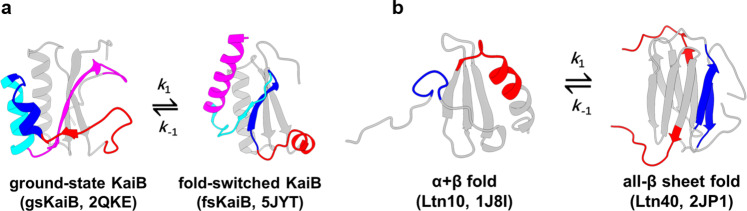
Metamorphic proteins undergo large-scale conformational rearrangements. **a** The interconversion between inactive ground-state KaiB (gsKaiB, PDB:2QKE) and active fold-switched KaiB (fsKaiB, PDB:5JYT). **b** The interconversion between inactive Ltn10-like XCL1 (PDB:1J8I) adopts α + β fold and active Ltn40-like XCL1 (PDB:2JP1) adopts all-β-sheet fold. The gray segments have no changes in secondary structure in both thermodynamically accessible states and the same color represents same primary structure but adopts distinct secondary structures in both states. *k*_1_ and *k*_-1_ correspond to the forward interconversion from inactive form (gsKaiB and Ltn10-like XCL1) to active form (fsKaiB and Ltn40-like XCL1) and the reverse interconversion from active form to inactive form, respectively.

Metamorphic proteins defy the classic protein folding paradigm “one sequence, one fold” proposed by Christian Anfinsen^1^ and demonstrate the ability to switch reversibly between generally two different native folds with entirely distinct functions^2^. Metamorphic proteins play vital roles in regulating biological processes in diverse life kingdoms, such as bacterial gene expression^3,4^, cyanobacterial circadian clock^5^ and human innate immune responses^6,7^. Unlike other well-known groups of proteins including: intrinsically disordered proteins (IDPs)^8^, morpheeins^9^, moonlighting proteins^10,11^ and prions^12^, metamorphic proteins have evolved to toggle between their alternative folds in physiological conditions by undergoing significant conformational changes such as the interconversion between the entire α-helices and β-strands (Fig. 1). Although only 20 proteins have been experimentally verified as metamorphic mostly through serendipity^13,14^, an increasing number of metamorphic proteins have been demonstrated in recent years and estimates from RCSB Protein Data Bank (PDB) searching indicate that 0.5-4% PDB proteins belong to this regime^15^.

All proteins execute their functions in a living cell or crowded environments, which is crowded by a bunch of macromolecules, such as proteins, nucleic acids, carbohydrates. For example, the concentration of macromolecules in *Escherichia coli* can reach up to 300-400 mg/ml and thus occupy the 20%-40% of cellular volume^16^. Consequently, this crowded environment can affect the folding^17,18^, protein kinetics and dynamics^19^, protein-protein interactions^20,21^, etc. To our knowledge, however, it is not clear how this crowded environment affects the interconversion between different structures of metamorphic proteins. In our work, two well characterized metamorphic proteins, KaiB and XCL1, have been chosen to study the impact of crowded conditions on the conformational switching that is responsible for performing distinct biological functions. KaiB, a core cyanobacterial circadian clock protein has been thus far found solely in circadian clock by LiWang Lab^5^ and involves in playing an important role in maintaining ~24 h circadian rhythm to synchronize with the timing of the earth’s rotation, flips between inactive ground-state KaiB (gsKaiB), which adopts homo-tetrameric βαββααβ fold, and active fold-switched KaiB (fsKaiB), which adopts monomeric βαβαββα thioredoxin-like fold that binds to fully phosphorylated KaiC (pS431;pT432) during night, to trigger the day-night transition of the circadian clock^22^. XCL1 (is also known as lymphotactin), is the human immune metamorphic protein and is a member of C chemokine sub-family, regulates the migration of leukocytes in response to inflammation by coupling its chemokine structure binds to the G-coupled protein receptor (GPCR) and its alternate dimeric all-beta structure binds to the glycosaminoglycans (GAGs)^6,7^.

Here, we measured the kinetics and thermodynamics of KaiB and XCL1 fold-switching in dilution conditions and in crowded conditions such as Ficoll400 and bovine serum albumin (BSA) or PEG10K. We demonstrated that crowded environments favor the formation of inactive states of KaiB and XCL1 without disturbing the corresponding structures comparing to in dilute conditions (Supplementary Fig. S1).

## Results

### The impact of crowded environments on protein metamorphosis occurs on timescales of seconds

**Fig. 2:**
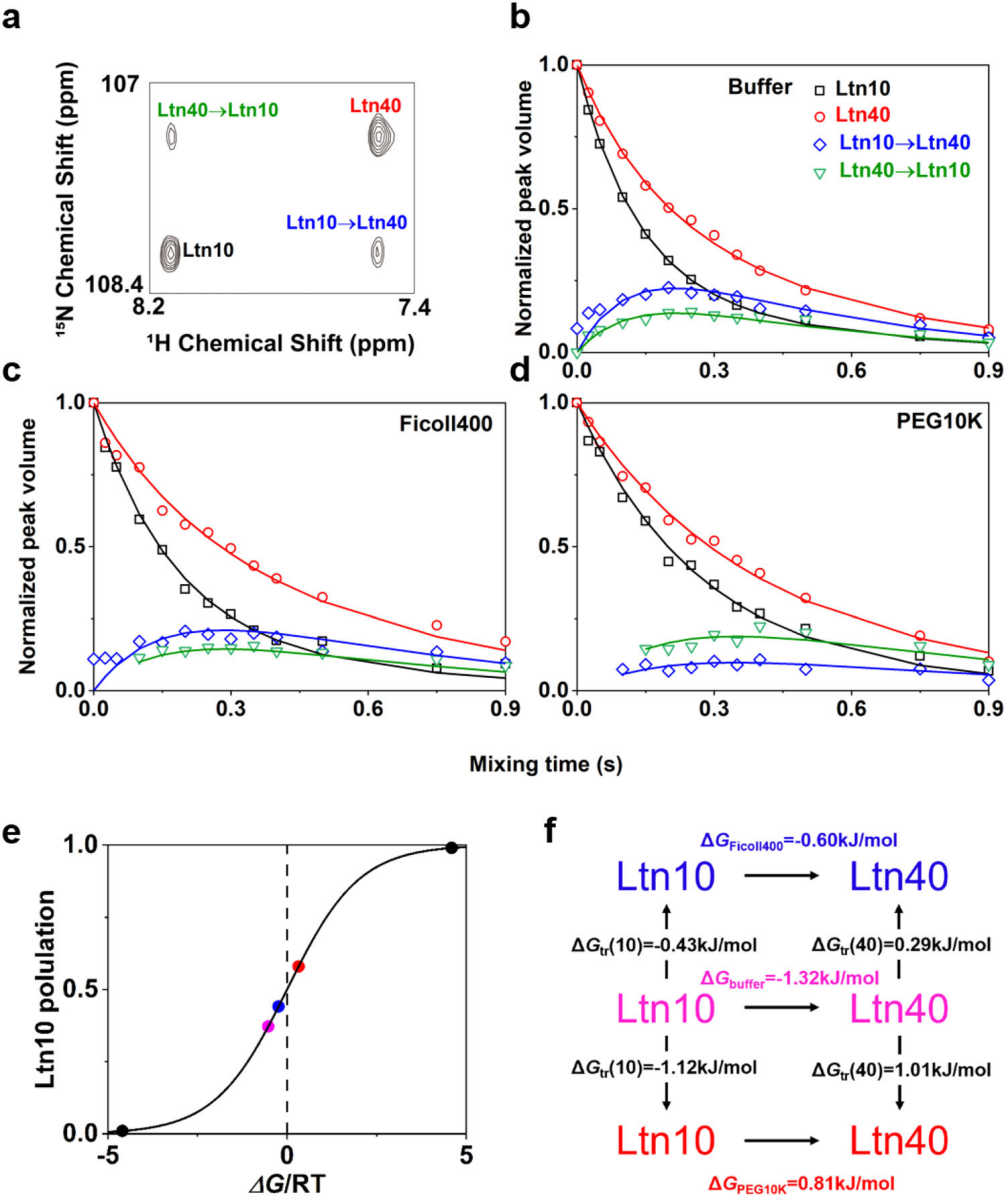
The kinetics and thermodynamics of XCL1 metamorphosis in dilute buffer and crowded conditions. **a** ZZ-exchange spectra for residue Gly44 of XCL1 at 40 °C. **b**–**d** Normalized ZZ-exchange peak volume plots for residue Gly44 of XCL1 in buffer (**b**), Ficoll400 (**c**) and PEG10K (**d**). Black squares and red circles correspond to the Ltn10-like and Ltn40-like XCL1, respectively. Blue diamonds and green triangles correspond to forward interconversion from Ltn10-like XCL1 to Ltn40-like XCL1 and reverse interconversion from Ltn40-like XCL1 to Ltn10-like XCL1, respectively. **e** The populations of Ltn10-like XCL1 were calculated from HSQC peak volume of residue Gly44 in buffer (magenta circle), Ficoll400 (blue circle) and PEG10K (orange circle) and are plotted as a function of the associated free energy, Δ*G*/RT, where R is the gas constant and T is the temperature. Black circles correspond to the population of Ltn10-like XCL1 is supposed to be 99% or 1%, respectively. The value of free energy (both XCL folds have same population) equals to zero is shown by the vertical dash line. **f** Transfer free energies (Δ*G*_tr_) driven interconversion from Ltn40-like XCL1 to Ltn10-like XCL1 in crowded conditions comparing to dilute buffer, positive and negative Δ*G*_tr_ values indicate that it is an Δ*G*_tr_-unfavorable and a Δ*G*_tr_-favorable shift, respectively, from dilute buffer to crowded condition.

In this work, we chose one metamorphic protein is the ancestor of XCL1, Anc.3 (hereafter, we refer to this ancestor as XCL1), which was resurrected by Volkman Lab^6^. XCL1 undergoes fold-switching on a time scale of seconds^6^, ZZ-exchange method^23,24^, which is referred to as magnetization exchange for probing biomolecular dynamics on slow timescales of seconds, was applied to measure XCL1 metamorphosis in dilute buffer and in crowded conditions, including Ficoll400 and PEG10K. Gly44 was chosen to be a probe to measure the kinetics, because it shows two peaks that correspond to the XCL1’s two different structures and undergoes interconversion between Ltn10-like fold with ^1^H chemical shift of ~8.1ppm and Ltn40-like fold with ^1^H chemical shift of ~7.5ppm (Fig. 2a). To understand the macromolecular crowding effect on XCL1 metamorphosis, we chose two popular crowding agents, Ficoll400 and PEG10K, to mimic the crowded intracellular environment. We then measured the kinetics of XCL1 metamorphosis in dilute buffer and crowded conditions. Under dilute buffer the exchange rate, *k*_ex_, has been determined to 4.81 ± 0.04 s^−1^ and 37 ± 0.1% of XCL1 occupies Ltn10-like fold (Fig. 2b, e, Supplementary Fig. S2 and Table 1). The addition of 90 g/L Ficoll400 to the buffer leads to the increased population of Ltn10-like XCL1 by ~7% resulting in Ltn10-like XCL1 populates 44 ± 0.1% with a slightly decreased exchange rate (*k*_ex_, 4.30 ± 0.38 s^−1^) (Fig. 2c, e, Supplementary Fig. S2 and Table 1). This increased population of Ltn10-like XCL1 (58 ± 4%) is also observed for the addition of 90 g/L PEG10K. Notably, the exchange rate decreases significantly by ~57% in the presence of 90 g/L PEG10K resulting in 2.08 ± 0.32 s^−1^ (Fig. 2d, e, Supplementary Fig. S2 and Table 1). Moreover, our data show that Ficoll400 and PEG10K also can give rise to pronounced reduction in the forward interconversion rate (*k*_1_ for Ficoll400, 2.41 ± 0.18 s^−1^; *k*_1_ for PEG10K, 0.87 ± 0.09 s^−1^), albeit both crowders cause subtle reduction or no changes in the reverse interconversion rate (*k*_-1_ for Ficoll400, 1.89 ± 0.20 s^−1^; *k*_-1_ for PEG10K, 1.21 ± 0.23 s^−1^) (Table 1). Transfer free energies (Δ*G*_tr_) act as indicators for empirically describing effective protein-crowder interactions^25^. The transfer free energy was calculated from the population ratio. Our data show that transfer free energy has opposite effects on the populations of Ltn10-like and Ltn-40-like XCL1 states. Positive Δ*G*_tr_ values of Ltn40-like XCL in crowded conditions indicate that effective protein-crowder interactions (hard-core repulsions and chemical interactions mainly include electrostatic interactions and hydrophobic interactions^20,21,26–29^) mitigate the formation of homo-dimeric Ltn40-like XCL1 in the addition of 90 g/L Ficoll400 and PEG10K, in contrast, negative Δ*G*_tr_ values indicate that this complicated interactions enhance the population of Ltn10-like XCL1 in crowded conditions (Fig. 2f and Table 1).

**Table 1:** Kinetic and thermodynamic parameters of protein metamorphism in dilute buffer and crowded conditions.

	KaiB^G89A^					
	*k*_ex_(h^-1^)	*P*_gs_	*P*_fs_	*k*_1_(h^-1^)	*k*_-1_(h^-1^)	Δ*G*(kJ/mol)
**Buffer**	0.44 ± 0.02	0.80 ± 0.01	0.2 ± 0.01	0.09 ± 0.01	0.35 ± 0.01	3.49 ± 0.16
**Ficoll400**	0.42 ± 0.01	0.85 ± 0.02	0.15 ± 0.02	0.06 ± 0.01	0.36 ± 0.02	4.37 ± 0.34
**BSA**	0.48 ± 0.05	0.93 ± 0.01	0.07 ± 0.01	0.03 ± 0.01	0.45 ± 0.04	6.52 ± 0.51
	**KaiB**^**D91R**^					
**Buffer**	0.28 ± 0.01	0.64 ± 0.01	0.36 ± 0.01	0.10 ± 0.01	0.18 ± 0.01	1.45 ± 0.05
**Ficoll400**	0.28 ± 0.01	0.64 ± 0.01	0.36 ± 0.01	0.10 ± 0.01	0.18 ± 0.01	1.45 ± 0.03
**BSA**	0.24 ± 0.01	0.71 ± 0.01	0.29 ± 0.01	0.07 ± 0.01	0.17 ± 0.01	2.26 ± 0.07
	**XCL1**					
**Buffer**	4.81 ± 0.04	0.37 ± 0.001	0.63 ± 0.001	3.03 ± 0.02	1.78 ± 0.01	−1.39 ± 0.006
**Ficoll400**	4.30 ± 0.38	0.44 ± 0.001	0.56 ± 0.001	2.41 ± 0.18	1.89 ± 0.20	−0.63 ± 0.006
**PEG10K**	2.08 ± 0.32	0.58 ± 0.04	0.42 ± 0.04	0.87 ± 0.09	1.21 ± 0.23	0.84 ± 0.04

### The impact of crowded environments on protein metamorphosis occurs on timescales of hours

**Fig. 3:**
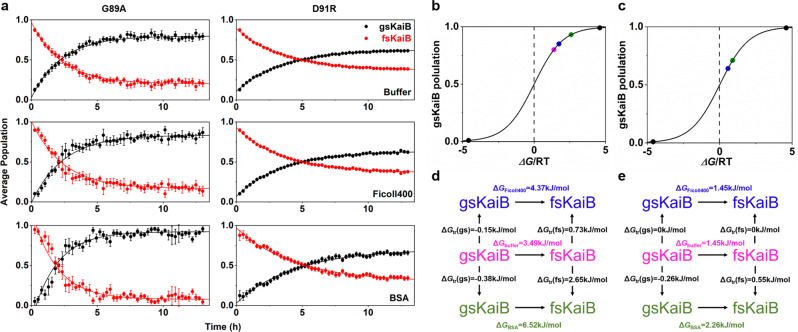
The kinetics and thermodynamics of KaiB metamorphosis in dilute buffer and crowded conditions. **a** The populations of gsKaiB (black dots) and fsKaiB (red dots) of KaiB^G89A^ (left) and KaiB^D91R^ (right) variants were calculated from averaged HSQC peak volumes of reporter residues in buffer (upper), Ficoll400 (middle) and BSA (bottom) and are plotted as a function of time. All kinetic parameters and thermodynamic parameters were extracted by fitting these time courses of averaged peak volumes. Data are shown as mean ± SEM. **b**, **c** The populations of gsKaiB of KaiB^G89A^ (**b**) and KaiB^D91R^ (**c**) variants are plotted as a function of the associated free energy in buffer (magenta circle), Ficoll400 (blue circle) and BSA (green circle). Black circles correspond to the population of gsKaiB is supposed to be 99% or 1%, respectively. **d**, **e** Transfer free energies driven interconversion from fsKaiB to gsKaiB in crowded conditions comparing to dilute buffer, positive and negative Δ*G*_tr_ values indicate that it is an Δ*G*_tr_-unfavorable and a Δ*G*_tr_-favorable shift, respectively, from dilute buffer to crowded condition.

The other metamorphic protein that we have been working on is the cyanobacterial circadian clock protein KaiB, which undergoes fold-switching on a time scale of hours^5^. Real-time NMR experiments were applied to monitoring the changing of populations of gsKaiB and fsKaiB over time in diluted buffer condition and concentrated crowded conditions (90 g/L Ficoll400 and 90 g/L BSA) (Fig. 3a and Supplementary Fig. S3). Here, we chose two KaiB mutants, KaiB^G89A^ and KaiB^D91R^, as the test proteins because the 2D ^1^H-^15^N heteronuclear single quantum coherence spectroscopy (HSQC) spectrum of each mutant presents two distinct sets of chemical shifts, which exist in a slow conformational equilibrium between fsKaiB and gsKaiB. Under dilute buffer the exchange rates, *k*_ex,_ have been determined to 0.44 ± 0.02 h^−1^ and 80 ± 1% of KaiB^G89A^ occupies gsKaiB fold and 0.28 ± 0.01 h^−1^ and 64 ± 1% of KaiB^D91R^ occupies gsKaiB fold (Fig. 3b, c and Table 1). The addition of 90 g/L Ficoll400 to the buffer leads to the increased population of gsKaiB of KaiB^G89A^ by ~5% resulting in gsKaiB populates 85 ± 1% with a slightly decreased exchange rate (*k*_ex_, 4.20 ± 0.01 h^-1^) (Fig. 3b and Table 1), but there is no effect on the population of gsKaiB and the exchange rate of KaiB^D91R^ in the presence of 90 g/L Ficoll400 (Fig. 3c and Table 1). The increased population of gsKaiB (93 ± 1% for KaiB^G89A^ and 71 ± 1% for KaiB^G91R^) is also observed for the addition of 90 g/L BSA. Moreover, our data show that the forward interconversion rates decrease significantly by ~33% and ~67% for KaiB^G89A^ (*k*_1_, 0.03 ± 0.01 h^-1^) and KaiB^D91R^ (*k*_1_, 0.07 ± 0.01 h^-1^), respectively, in the presence of 90 g/L BSA, albeit BSA only causes pronounced increment in the reverse interconversion rate of KaiB^G89A^ (*k*_-1_, 0.45 ± 0.04 h^-1^) and negligible changes in the reverse interconversion rate of KaiB^D91R^ (Table 1). Furthermore, the transfer free energies in 90 g/L Ficoll400 and BSA were also calculated. The Δ*G*_tr_-unfavorable shift (positive Δ*G*_tr_) of fsKaiB and Δ*G*_tr_-favorable shift (negative Δ*G*_tr_) of gsKaiB, respectively, from dilute buffer to crowded conditions, were observed (Fig. 3d).

### The impact of crowded environments on free-energy landscape of metamorphic proteins

**Fig. 4:**
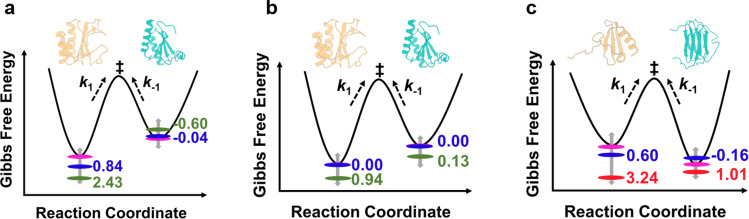
Free energy landscapes of protein metamorphosis in dilute buffer and crowded conditions. The values correspond to the energy barrier differences ($\varDelta \varDelta {G}^{{\ddagger} }=\varDelta {G}_{{{{\rm{crowded}}}}}^{{\ddagger} }-\varDelta {G}_{{{{\rm{buffer}}}}}^{{\ddagger} }$) of KaiB mutants (**a**, KaiB^G89A^; **b**, KaiB^D91R^) and XCL1 (**c**) between buffer (magenta) and 90 g/L Ficoll400 (blue), 90 g/L BSA (green) or 90 g/L PEG10K (red).

The effect of crowding on the kinetics (rate) and thermodynamics (population) can be better illustrated in a free energy diagram (Fig. 4), and many studies have demonstrated that the crowded conditions can significantly reshape the energy landscapes of tested proteins^28,30–34^. The difference of energy barrier in dilute buffer and crowded condition was calculated by using Eyring equation. Here, the transition state is used to be a reference, for KaiB^G89A^ mutant, the addition of 90 g/L Ficoll400 results in the decreased free energy (increased energy barrier, $\varDelta {G}^{{\ddagger} }$) of gsKaiB, which is consistent with other studies that hard-core repulsions arising from crowders favor a compact state^35,36^ of protein, as well as results in a negligible increase in free energy of fsKaiB. Interestingly, there are no perturbations in free energies of both KaiB states for KaiB^D91R^ mutant in presence of 90 g/L Ficoll400, this is more likely that the charged residue substitution has already changed the way that KaiB communicates with its surrounding crowders through a combination of excluded volume effect and “soft” chemical interaction^37–39^. There are also observable decreases in free energies of gsKaiB states of both KaiB mutants in the presence of 90 g/L BSA as compared with diluted buffer, indicating that more negatively charged residues exposed in gsKaiB state is involved in the repulsive electrostatic interaction with the negatively charged BSA (pI, 4.7) at pH 7.0, which in turn stabilizes the gsKaiB state, however, the attractive electrostatic interaction between fsKaiB with more positively charged surfaces and BSA destabilizes the fsKaiB state and thereby resulting in an increase in the free energy of fsKaiB for KaiB^G89A^ mutant (Fig. 4a, b and Supplementary Fig. S4). The surprising decreased free energy of fsKaiB for KaiB^D91R^ mutant, even with the value of ca. 0.13 kJ/mol, can be ignored more likely because of the slightly decreased reverse rate was used for energy barrier calculation is within error (Table 1). For chemokine XCL1, crowding agents, 90 g/L Ficoll400 and PEG10K, stabilize the Ltn10-like fold because of the smaller van der Waals volume and solvent-accessible surface area (SASA) of Ltn10-like XCL1 allow it to occupy the limited accessible space that excluded by crowders (Supplementary Table S1).

## Conclusions

In vitro investigations of protein structure, dynamics and function are usually performed in dilute buffer. However, these highly dilute environments, are dramatically different from the crowded and complicated intracellular environments in which proteins carry out their functions. Consequently, the dense environment affects protein dynamics, stability, folding, enzymatic activity, etc. In this study, our work demonstrates the impact of macromolecular crowding on protein metamorphosis, and also suggests that the more crowded and complicated environments in cell have significant effects on the fold-switching of metamorphic proteins and then modulate their functions in response to environmental stimuli. Our observations pave the way for future in-cell studies of protein metamorphosis and improving the understanding of the function of metamorphic proteins within cell.

## Methods

### Protein expression and purification

All KaiB mutants were expressed and purified using previously published methods^5^. All protein samples were prepared in standard M9 minimal media containing ^15^N-labeled ammonium chloride (Cambridge Isotope Laboratories, Inc. (CIL)). In brief, cells were grown to OD_600_ = ~ 0.6 in M9 minimal medium and induced by adding final isopropyl β-d-1-thiogalactopyranoside (IPTG) concentration of 200 µM for 12 h at 30 °C. Cells were harvested and cell pellets were resuspended using lysis buffer (50 mM NaH_2_PO_4_, 500 mM NaCl, pH 8.0) and homogenized with an Avestin C3 Emulsiflex homogenizer (Avestin Inc, Canada). Loaded supernatant onto Ni-NTA column (QIAGEN, #30230) and washed with wash buffer (50 mM NaH_2_PO_4_, 500 mM NaCl, 20 mM imidazole, pH 8.0) following centrifugation, eluted His-tagged KaiB by adding elution buffer (50 mM NaH_2_PO_4_, 500 mM NaCl, 250 mM imidazole, pH 8.0). Added His-ULP1 protease to final concentration of 3 µM for cleavage for 12 h followed by loading 10-times diluted digested sample onto Ni-NTA column to remove the SUMO tag. Finally, SDS-PAGE analysis of protein purity following applying FPHC with HiLoad 16/600 Superdex 75 size-exclusion column in phosphate buffer (20 mM Na_2_HPO_4_/NaH_2_PO_4_, 100 mM NaCl, pH 7.0).

The gene sequence encoding XCL1 was synthesized by Tsingke Biotechnology Co., Ltd., which contains a thioredoxin tag and 6x-His tag followed by an enterokinase cutting site on the N terminal of XCL1. The XCL1 plasmid was transformed into E. coli BL21(DE3) competent cells (Novagen) and expressed in minimal media with ^15^NH_4_Cl as sole nitrogen source. The cells were grown at 37 °C, and when OD_600_ reached to the value of ~ 0.7, the isopropyl β-D-1-thiogalactopyranoside (IPTG) was added into the cell culture flask with the final concentration of 0.5 mM and shook at 220 rpm at 22 °C for 16 hours. After that, the cells were harvested by centrifugation at 4400 × g, 4 °C for 10 min, and the supernatant was decanted.

The cell pellet was then resuspended in buffer A (6 M Guanidine HCl, 200 mM NaCl, 50 mM Tris, pH 8.0) with 10 mM benzamidine added. Lysing cells was carried out by passing through of a French press twice at 16,000 p.s.i followed with centrifuged at 27,000 × g for 1 hour at 4 °C. The supernatant which contained the protein of interest was collected.

The supernatant was loaded onto a buffer A balanced home packed nickel chelating column (Qiagen) three times. After that, the column was washed with 10 column volumes of buffer A, and then buffer B (6 M Guanidinium chloride, 200 mM NaCl, 80 mM NaPi, pH 7.2). Proteins were then eluted from the column with buffer C (6 M guanidine hydrochloride, 200 mM NaCl, and 60 mM NaOAc, pH 4). The fractions were combined after verification from SDS-PAGE gel, and then brought pH to 8.0 followed with 10 mM βME added into the protein solution and stirring at room temperature for 2 hours. The proteins were then dripped into 10 × volume of ice-cold refolding buffer (550 mM L-arginine hydrochloride, 400 mM sucrose, 9.6 mM NaCl, 0.4 mM KCl, 2 mM CaCl2, 2 mM MgCl2, 2 mM reduced glutathione (GSH), 0.2 mM oxidized glutathione (GSSG), 50 mM Tris, pH 8.0) while stirring and allowed the protein in refolding buffer for 24 hours at 4 °C while stirring. After refolding, the proteins were dialyzed against 4 liters of dialysis buffer (200 mM NaCl, 20 mM Tris, pH 7.5) for 4 times.

The proteins in dialysis bags were then poured into a clean beaker with 2 mM CaCl_2_ and 650 nM enterokinase were added into the protein solution, and cleaved for 3 days at 4 °C. After cleavage, same volume of Milli-Q water was added to the protein solution followed with adding 0.2% of TFA and 10% C4 buffer B (100% acetonitrile, 0.1% TFA). Protein solution was filtered (0.45 μm) before loading to a C4 reversed-phase chromatography column. The target protein was then eluted out with an acetonitrile gradient (C4 buffer A: 100% H2O 0.1% TFA, C4 buffer B: 100% acetonitrile, 0.1% TFA). The fractions with protein of interest were lyophilized for further usage.

### NMR spectroscopy

All experiments were performed on a Bruker Avance III 600 MHz spectrometer equipped with a TCI cryoprobe. ^1^H chemical shifts were referenced to internal DSS and ^15^N chemical shifts were indirectly referenced to DSS using absolute frequency ratios listed on the BMRB website^40^. All NMR data were processed with NMRPipe^41^ and analyzed using nmrDraw or NMRFAM-Sparky^42^. In order to mimic the crowded environments, the commonly used crowders, Ficoll400, PEG10K and BSA, were chosen for this study. PEG and Ficoll are the most popular macromolecular crowding agents that have been used to mimic the intracellular environments, these polymers are highly soluble and inert macromolecules with neutral charge, in this study, the PEG10K (M.W. ~10,000) and Ficoll400 (M.W. ~400,000) were used. BSA (pI 4.7, M.W. ~66 kDa) also is the commonly used bio-macromolecules to mimic intracellular congestion.

For real-time NMR experiments, all experiments were performed at 30 °C. NMR samples contained 200 μM ^15^N-enriched KaiB protein in 350 μL NMR buffer (20 mM Na_2_HPO_4_/NaH_2_PO_4_, 100 mM NaCl, 0.02% NaN_3_,10 μM DSS, pH 7.0, 90% H_2_O/10% D_2_O). The crowder power were added to the NMR sample and the pH of the resulting solution has negligible pH changes (<0.1 pH units) after addition of crowders. Experiments were performed in triplicate. The spectra were collected in a [F1, F2] spectral widths of [1583.901 Hz, 9615.385 Hz] with 1306 × 200 complex points, recycle delay of 1.0 s and [F1, F2] transmitter frequency offset of [2824 Hz, 7248.52 Hz]. The peak volumes were used for calculating the population of each KaiB state. Exchange rates were determined by fitting of population of each KaiB state as a function of time using the following equations, in which *k*_1_ and *k*_-1_ are the forward rate and reverse rate, respectively, and *k*_ex_ = *k*_1_ + *k*_-1_, [gsKaiB]_0_/[fsKaiB]_0_ and [gsKaiB]_t_/[fsKaiB]_t_ are the initial populations and the populations at each timepoint.

$${[{{{\rm{gsKaiB}}}}]}_{t}=\frac{{k}_{-1}}{{k}_{{{{\rm{ex}}}}}}+\left({[{{{\rm{gsKaiB}}}}]}_{0}-\frac{{k}_{-1}}{{k}_{{{{\rm{ex}}}}}}\right)\exp (-{k}_{{{{\rm{ex}}}}}{{{\rm{t}}}})$$

$${[{{{\rm{fsKaiB}}}}]}_{t}=\frac{{k}_{1}}{{k}_{{{{\rm{ex}}}}}}+\left({[{{{\rm{fsKaiB}}}}]}_{0}-\frac{{k}_{1}}{{k}_{{{{\rm{ex}}}}}}\right)\exp (-{k}_{{{{\rm{ex}}}}}{{{\rm{t}}}})$$

^1^H-^15^N ZZ-exchange experiments were applied to ~1 mM ^15^N XCL1 in 350 μL NMR buffer (20 mM Na_2_HPO_4_/NaH_2_PO_4_, pH 6.5, 0.02% NaN_3_,10 μM DSS, 90% H_2_O/10% D_2_O) with different mixing times (0, 25, 50, 100, 150, 200, 250, 300, 350, 400, 500, 750 and 900 ms) at 40 °C. Experiments were performed in duplicate. Peak volumes of auto and cross peaks for each mixing time were extracted using NMRDraw. All peak volumes were normalized the auto peaks at mixing time equal to 0. Exchange rates were determined by fitting of peak volumes as a function of mixing time using the following equations^43^, in which refer to the volumes for the Ltn10-like XCL1 (A), Ltn-40 like XCL1 (B), Ltn10→Ltn40 (A → B) and Ltn40→Ltn10 (B → A). The values in Table 1 shown are averages of two or three independent experiments ± SD. *k*_1_ and *k*_-1_ are the forward rate and reverse rate, respectively. *R*_1_ donates the relaxation rate.

$${I}^{{{{\rm{A}}}}}(t)=\frac{{I}_{0}^{{{{\rm{A}}}}}}{2}\left[\left(1-\frac{{R}_{1}^{{{{\rm{A}}}}}-{R}_{1}^{{{{\rm{B}}}}}+{k}_{1}-{k}_{-1}}{{\lambda }_{+}-{\lambda }_{-}}\right)\exp (-{\lambda }_{-}t)+\left(1+\frac{{R}_{1}^{{{{\rm{A}}}}}-{R}_{1}^{{{{\rm{B}}}}}+{k}_{1}-{k}_{-1}}{{\lambda }_{+}-{\lambda }_{-}}\right)\exp (-{\lambda }_{+}t)\right]$$

$${I}^{{{{\rm{B}}}}}(t)=\frac{{I}_{0}^{{{{\rm{B}}}}}}{2}\left[\left(1+\frac{{R}_{1}^{{{{\rm{A}}}}}-{R}_{1}^{{{{\rm{B}}}}}+{k}_{1}-{k}_{-1}}{{\lambda }_{+}-{\lambda }_{-}}\right)\exp (-{\lambda }_{-}t)+\left(1-\frac{{R}_{1}^{{{{\rm{A}}}}}-{R}_{1}^{{{{\rm{B}}}}}+{k}_{1}-{k}_{-1}}{{\lambda }_{+}-{\lambda }_{-}}\right)\exp (-{\lambda }_{+}t)\right]$$

$${I}^{{{{\rm{A}}}}\to {{{\rm{B}}}}}(t)={I}_{0}^{{{{\rm{A}}}}}\frac{{k}_{1}}{{\lambda }_{+}-{\lambda }_{-}}[\exp (-{\lambda }_{-}t)-\exp (-{\lambda }_{+}t)]$$

$${I}^{{{{\rm{B}}}}\to {{{\rm{A}}}}}(t)={I}_{0}^{{{{\rm{B}}}}}\frac{{k}_{-1}}{{\lambda }_{+}-{\lambda }_{-}}[\exp (-{\lambda }_{-}t)-\exp (-{\lambda }_{+}t)]$$

$${\lambda }_{\pm }=\frac{1}{2}\left[{R}_{1}^{{{{\rm{A}}}}}\,+\,{{R}}_{1}^{{{{\rm{B}}}}}\,+\,{k}_{1}\,+\,{k}_{-1}\pm \sqrt{{({R}_{1}^{{{{\rm{A}}}}}-{R}_{1}^{{{{\rm{B}}}}}+{k}_{1}-{k}_{-1})}^{2}+4{k}_{1}{k}_{-1}}\right]$$

The free energy barrier was calculated based on the transient state theory by using Eyring equation.

$${{\mathrm{ln}}}\,\frac{k}{{{{\rm{T}}}}}\,=\,-\frac{\varDelta {H}}{{R}{{{\rm{T}}}}}+\,{{\mathrm{ln}}}\,\frac{{k}_{{{{\rm{b}}}}}}{{{{\rm{h}}}}}+\frac{\varDelta {S}}{R}\,=\,{{\mathrm{ln}}}\,\frac{{k}_{{{{\rm{b}}}}}}{{{{\rm{h}}}}}-\frac{\varDelta {H}-{{{\rm{T}}}}\varDelta {S}}{{R}{{{\rm{T}}}}}=\,{{\mathrm{ln}}}\,\frac{{k}_{{{{\rm{b}}}}}}{{{{\rm{h}}}}}-\frac{\varDelta {{G}}^{{\ddagger} }}{{R}{{{\rm{T}}}}}$$

$$\varDelta \varDelta {G}^{{\ddagger} }=\varDelta {G}_{{{{\rm{crowded}}}}}^{{\ddagger} }-\varDelta {G}_{{{{\rm{buffer}}}}}^{{\ddagger} }=-R{{{\rm{T}}}}\,{{\mathrm{ln}}}\left(\frac{{k}_{{{{\rm{crowded}}}}}}{{k}_{{{{\rm{buffer}}}}}}\right)$$

### Reporting summary

Further information on research design is available in the Nature Portfolio Reporting Summary linked to this article.

## Supplementary information

Supplemental Information

Description of Additional Supplementary Files

Supplementary Data 1

Supplementary Data 2

Reporting Summary

## Data Availability

Protein structures used in this study were obtained from the RCSB Protein Data Bank (https://www.rcsb.org). PDB IDs are: ground-state KaiB (PDB:2QKE), fold-switched KaiB (PDB:5JYT), Ltn10-like XCL1 (PDB:1J8I) and Ltn40-like XCL1 (PDB:2JP1). Source data are provided with this paper. NMR spectra are provided in Supplementary Data 1 file. All raw data for studying the kinetics of fold switching of XCL1 and KaiB are provided in Supplementary Data 2 file.
