# Supplementary material for: Crowded environments tune the fold-switching in metamorphic proteins
Source: Commun Chem. 2023 Jun 8;6:117. doi: 10.1038/s42004-023-00909-2 (PMC10250422; doi:10.1038/s42004-023-00909-2)
Supplement: Supplementary file 1 — Supplemental Information [file 42004_2023_909_MOESM1_ESM.pdf]

Crowded environments tune the fold-switching in metamorphic proteins

Ning Zhang[1,2,3,§]*, Wenyan Guan[4,§], Shouqi Cui[1,2,3,5], Nana Ai[4]

Authors Institutional Affiliations

[1]Qingdao Institute of Bioenergy and Bioprocess Technology, Chinese Academy of Sciences, Qingdao 266101, China

[2]Shandong Energy Institute, Qingdao 266101, China

[3]Qingdao New Energy Shandong Laboratory, Qingdao 266101, China

[4]Materials and Biomaterials Science and Engineering, University of California, Merced, CA 95343, USA

[5]University of Chinese Academy of Sciences, Beijing, 100049, China

***Corresponding Author**

Qingdao Institute of Bioenergy and Bioprocess Technology, Chinese Academy of Sciences; Shandong Energy Institute; Qingdao New Energy Shandong Laboratory, Qingdao 266101, China

Ning Zhang

Email: zhangn2022@qibebt.ac.cn

ORCID ID: 0000-0002-3590-3774

[§]Ning Zhang and Wenyan Guan contributed equally.

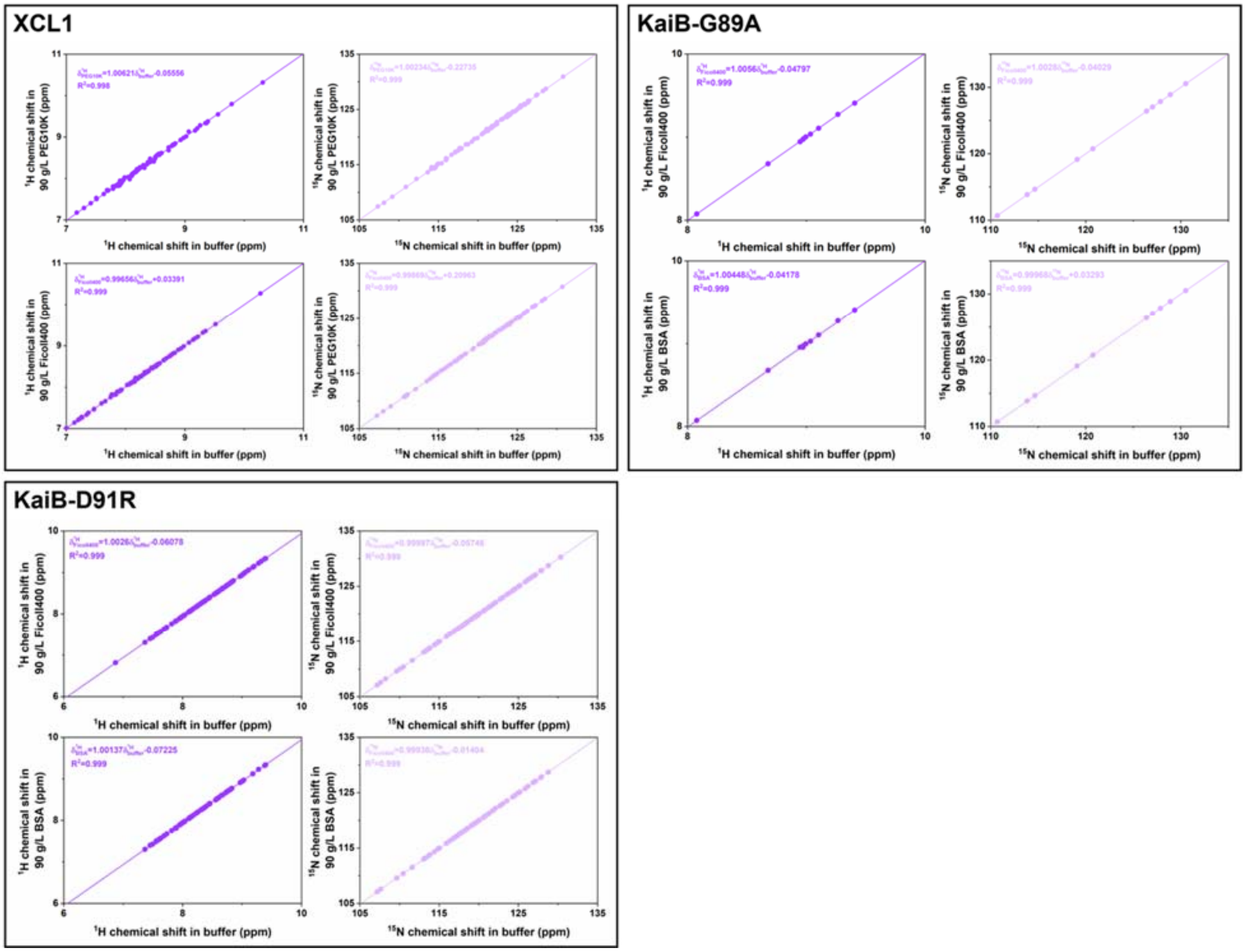

Fig. S1 | ^{1}H and ^{15}N Chemical shifts comparisons. Perfect linear regression (all slopes equal to ca. 1) between ^{1}H (^{15}N) chemical shifts in buffer and that in crowded conditions. Dark purple and light purple are corresponding to the ^{1}H chemical shift comparison and ^{15}N chemical shifts comparison, respectively.

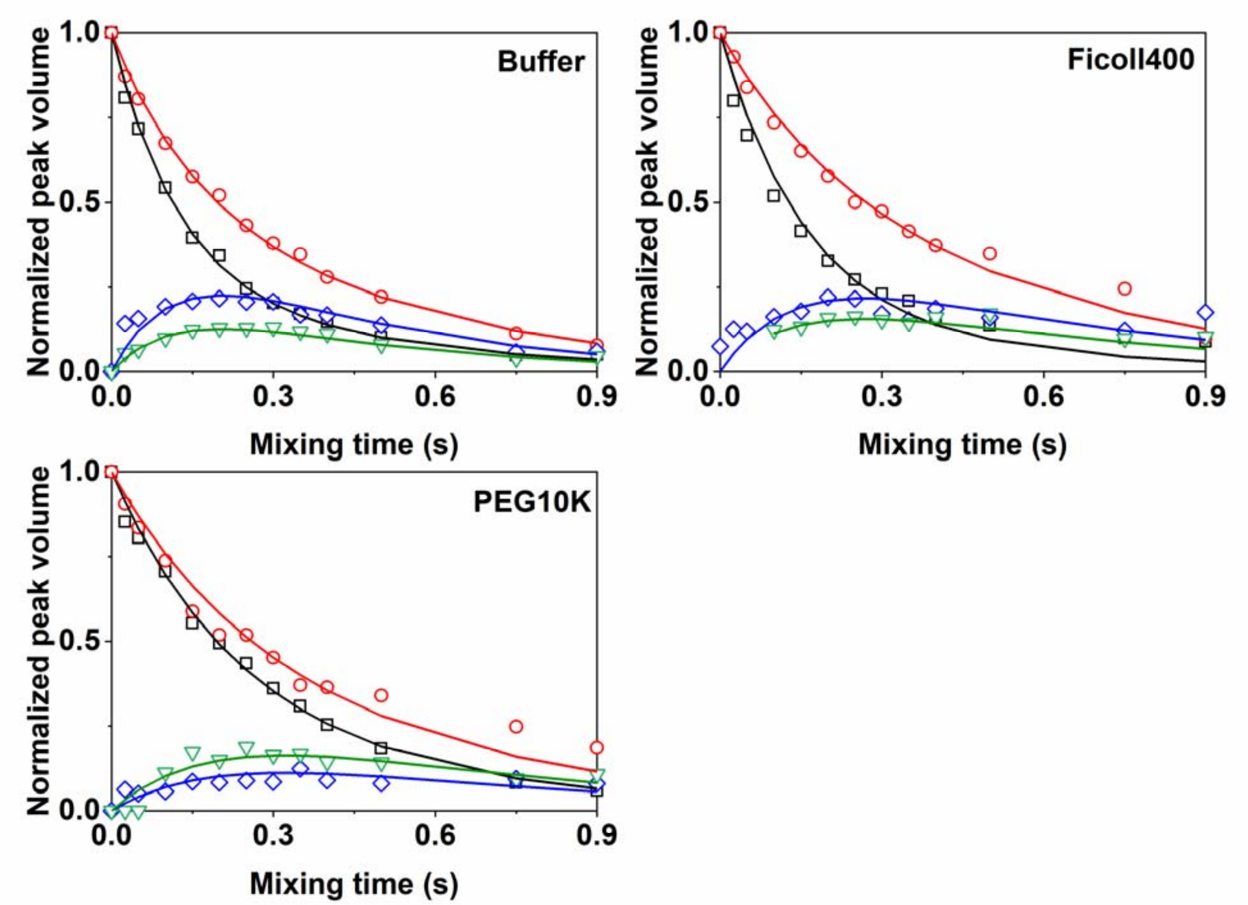

Fig. S2 | The kinetics and thermodynamics of XCL1 metamorphosis in dilute buffer and crowded conditions. Normalized ZZ-exchange peak volume plots for residue Gly44 of XCL1 in buffer, Ficoll400 and PEG10K. Black squares and red circles correspond to the Ltn10-like and Ltn40-like XCL1, respectively. Blue diamonds and green triangles correspond to forward interconversion from Ltn10-like XCL1 to Ltn40-like XCL1 and reverse interconversion from Ltn40-like XCL1 to Ltn10-like XCL1, respectively.

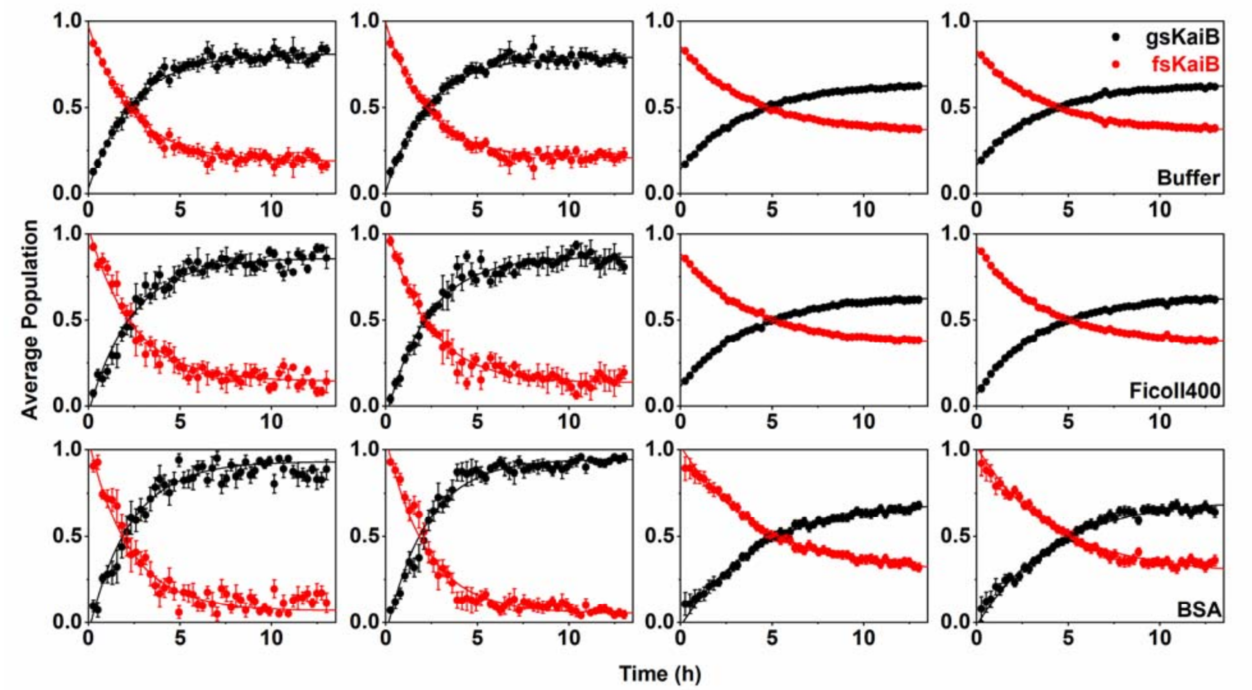

Fig. S3 | The kinetics and thermodynamics of KaiB metamorphosis in dilute buffer and crowded conditions. a, The populations of gsKaiB (black dots) and fsKaiB (red dots) of KaiB^{G89A} (left two columns) and KaiB^{D91R} (right two columns) variants were calculated from averaged HSQC peak volumes of reporter residues in buffer (upper), Ficoll400 (middle) and BSA (bottom) and are plotted as a function of time. All kinetic parameters and thermodynamic parameters were extracted by fitting these time courses of averaged peak volumes.

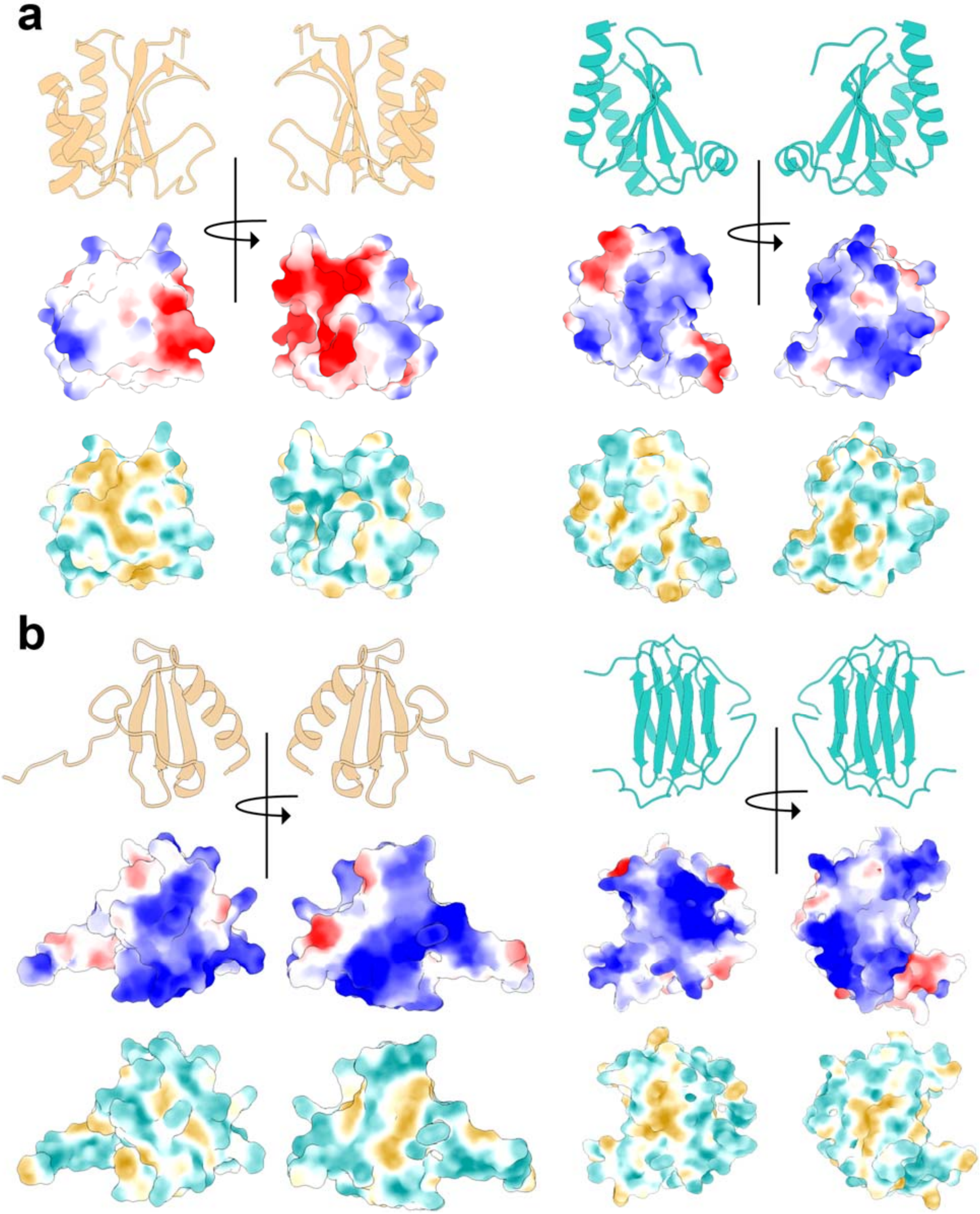
a
b

Fig. S4 | The electrostatic and hydrophobic analysis of two native states of metamorphic KaiB (a) and XCL1 (b). a, top row: the ribbon diagram of gsKaiB (left, PDB ID: 2QKE) and fsKaiB (right, PDB ID: 5JYT); middle row: the electrostatic potential analysis of both KaiB states. Red for negative potential through the white to blue for positive potential; bottom row: the hydrophobic analysis of both KaiB states. Dark cyan corresponds to the most hydrophilic zone to white to dark golden represents most hydrophobic surface. **b**, top row: the ribbon diagram of Ltn10-like XCL1 (left, PDB ID: 1J8I) and Ltn40-like XCL1 (right, PDB ID: 2JP1); middle row: the electrostatic potential analysis of both XCL1 states, red for negative potential through the white to blue for positive potential; bottom row: the hydrophobic analysis of both XCL1 states. Dark cyan corresponds to the most hydrophilic zone to white to dark golden represents most hydrophobic surface. All calculations were carried out in ChimeraX.

Table S1 | van der Waals volume calculation and solvent-accessible surface area calculation

GETAREA[1]			
Ltn10-like XCL1 (1J8I)	5334.51 Å^2	gsKaiB (2QKE)	6719.45 Å^2
Ltn40-like XCL1 (2JP1)	7698.96 Å^2	fsKaiB (5JYT)	6635.17 Å^2
ProteinVolume[2]			
Ltn10-like XCL1 (1J8I)	6980.78 Å^3	gsKaiB (2QKE)	11019.23 Å^3
Ltn40-like XCL1 (2JP1)	12055.63 Å^3	fsKaiB (5JYT)	10193.09 Å^3

Reference

1. Fraczkiewicz R, Braun W. Exact and efficient analytical calculation of the accessible surface areas and their gradients for macromolecules. *Journal of Computational Chemistry* **19**, 319-333 (1998).
2. Chen CR, Makhatadze GI. ProteinVolume: calculating molecular van der Waals and void volumes in proteins. *BMC Bioinformatics* **16**, 101 (2015).
