# Supplementary material for: Crowded environments tune the fold-switching in metamorphic proteins
Source: Commun Chem. 2023 Jun 8;6:117. doi: 10.1038/s42004-023-00909-2 (PMC10250422; doi:10.1038/s42004-023-00909-2)
Supplement: Supplementary file 2 — Description of Additional Supplementary Files [file 42004_2023_909_MOESM2_ESM.pdf]

Description of Additional Supplementary Files

File name: Supplementary Data 1

Description: 2D 1H-15N HSQC spectra of KaiBG89A, KaiBD91R and XCL1

File name: Supplementary Data 2

Description: All raw data for studying the kinetics of foldswitching of XCL1 and KaiB
