# Supplementary figures and images for: Crowded environments tune the fold-switching in metamorphic proteins
Source: Commun Chem. 2023 Jun 8;6:117. doi: 10.1038/s42004-023-00909-2 (PMC10250422; doi:10.1038/s42004-023-00909-2)

2D  $^1\text{H}$ - $^{15}\text{N}$  HSQC spectrum

KaiB<sup>G89A</sup>

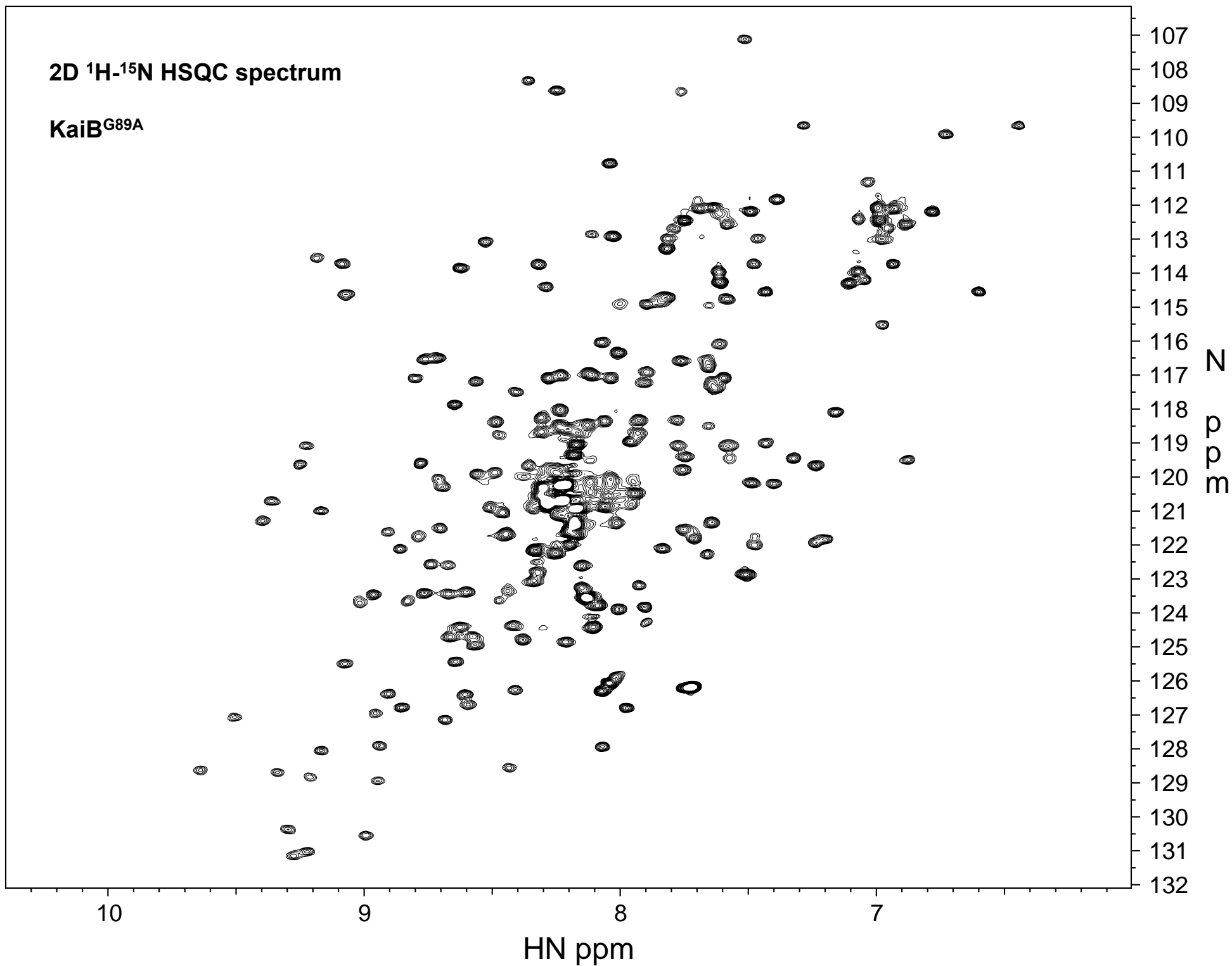

2D  $^1\text{H}$ - $^{15}\text{N}$  HSQC spectrum

KaiB<sup>D91A</sup>

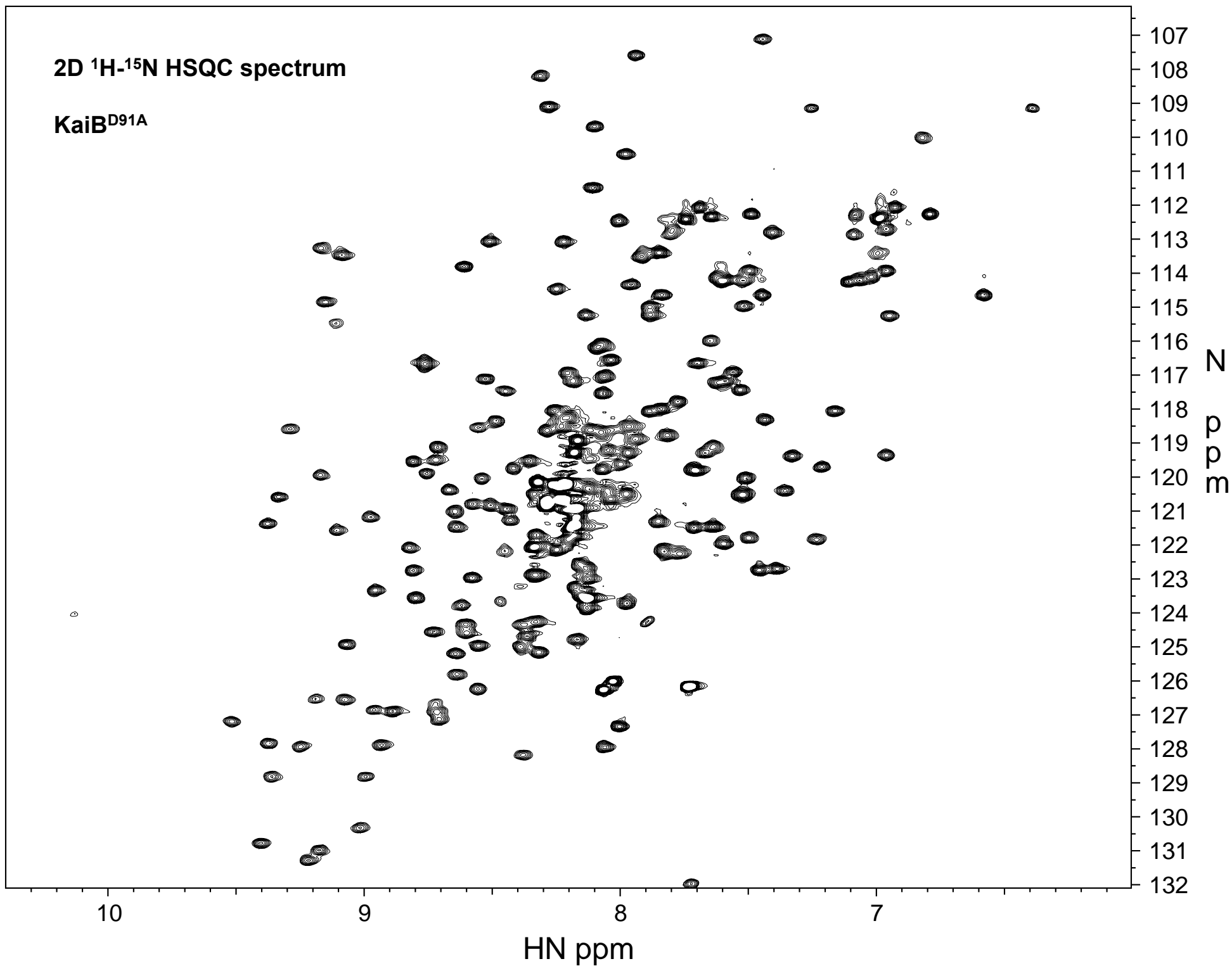

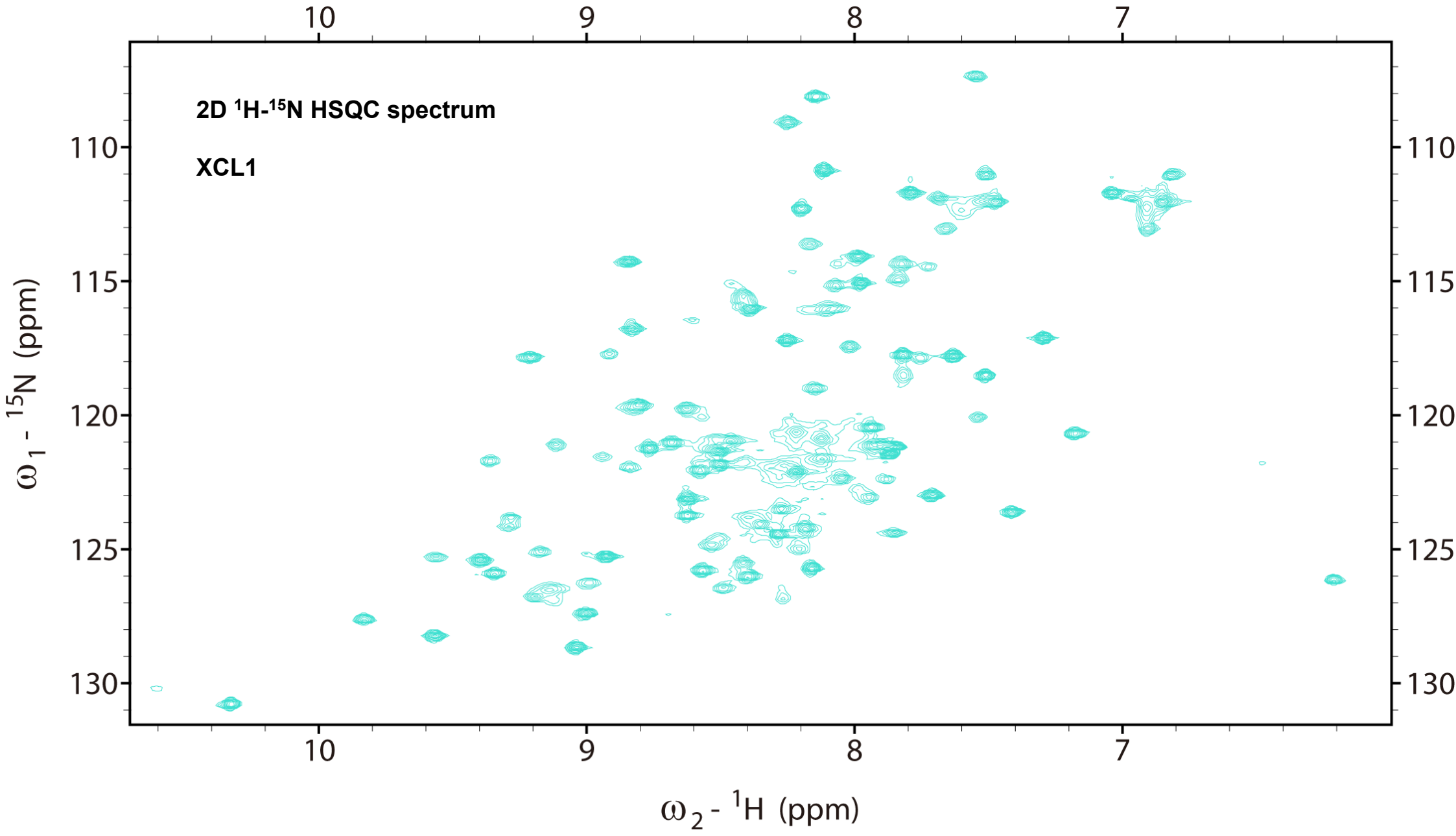

Supplement: Supplementary file 3 — Supplementary Data 1 [file 42004_2023_909_MOESM3_ESM.pdf]
